# Transforming Wound Management: Nanomaterials and Their Clinical Impact

**DOI:** 10.3390/pharmaceutics15051560

**Published:** 2023-05-22

**Authors:** Ashwini T, Ashlesh Prabhu, Vishal Baliga, Shreesha Bhat, Siddarth T. Thenkondar, Yogendra Nayak, Usha Y. Nayak

**Affiliations:** 1 Department of Pharmaceutics, Manipal College of Pharmaceutical Sciences, Manipal Academy of Higher Education, Manipal 576104, Karnataka, India; ashwini.t1@learner.manipal.edu (A.T.);; 2 Department of Pharmacology, Manipal College of Pharmaceutical Sciences, Manipal Academy of Higher Education, Manipal 576104, Karnataka, India; yogendra.nayak@manipal.edu

**Keywords:** wound care, bandages, nanomaterials, nanofibers, nanocomposites, silver-based nanoparticles, lipid nanoparticles, polymeric nanoparticles

## Abstract

Wound healing is a complex process that can be further complicated in chronic wounds, leading to prolonged healing times, high healthcare costs, and potential patient morbidity. Nanotechnology has shown great promise in developing advanced wound dressings that promote wound healing and prevent infection. The review article presents a comprehensive search strategy that was applied to four databases, namely Scopus, Web of Science, PubMed, and Google Scholar, using specific keywords and inclusion/exclusion criteria to select a representative sample of 164 research articles published between 2001 and 2023. This review article provides an updated overview of the different types of nanomaterials used in wound dressings, including nanofibers, nanocomposites, silver-based nanoparticles, lipid nanoparticles, and polymeric nanoparticles. Several recent studies have shown the potential benefits of using nanomaterials in wound care, including the use of hydrogel/nano silver-based dressings in treating diabetic foot wounds, the use of copper oxide-infused dressings in difficult-to-treat wounds, and the use of chitosan nanofiber mats in burn dressings. Overall, developing nanomaterials in wound care has complemented nanotechnology in drug delivery systems, providing biocompatible and biodegradable nanomaterials that enhance wound healing and provide sustained drug release. Wound dressings are an effective and convenient method of wound care that can prevent wound contamination, support the injured area, control hemorrhaging, and reduce pain and inflammation. This review article provides valuable insights into the potential role of individual nanoformulations used in wound dressings in promoting wound healing and preventing infections, and serves as an excellent resource for clinicians, researchers, and patients seeking improved healing outcomes.

## 1. Introduction

A wound can be defined as a disruption in the skin’s protective function, characterized by the loss of integrity and cohesiveness of the epithelium, with or without damage to underlying connective tissue [1]. This damage can occur as a result of physical trauma or thermal injury. Wounds can be categorized as acute or chronic based on the underlying healing process and duration. Acute wounds typically arise from sudden trauma or surgical procedures, and they tend to follow a predictable healing course, which typically takes between 8 to 12 weeks to complete [1,2]. The duration of the healing process is influenced by the extent of damage sustained by the various skin layers [3].

In contrast, chronic wounds, such as those arising from burns, decubitus ulcers, and leg ulcers, lack a defined healing timeframe and may require specialized management approaches to promote healing [4]. Chronic wounds affect about 1–2% of the population in Europe and the United States, with diabetes patients being particularly susceptible [2]. Even though various traditional therapies are available, they are expensive, prolonged, and have a high rate of ulcer relapse [5]. The large number of patients seeking improved healing outcomes, coupled with the increasing cost of wound care, has prompted research into wound healing and skin regeneration [6,7].

Nanotechnology has led to the development of nano-drug delivery systems that have shown promising results in skin regeneration [8]. Nano-drug delivery systems are compatible with the skin, creating a favorable moist environment for wound healing [9,10]. Specific nano-drug delivery systems can enter the cytoplasmic space or activate specific transport mechanisms, improving drug retention [11,12,13,14,15]. Incorporating bioactive molecules protects drugs from degradation and enhances therapeutic effectiveness. Sustained drug release prolongs effective drug concentration, reducing administration frequency and improving compliance while reducing costs [14,16].

The development of nanomaterials in wound care has complemented the use of nanotechnology in drug delivery systems [10,17,18,19]. Nanomaterials are one of the most widely used materials in wound care to address the complexities of the normal wound healing cycle, cell-type specificity, a surplus of regulating molecules, and chronic wound pathophysiology [11,20]. By using biocompatible and biodegradable nanomaterials, drug delivery systems can be designed to enhance wound healing and provide sustained drug release. The application of wound dressings offers a range of benefits, including preventing wound contamination by securing the dressing in place [21,22]. Dressings also support the injured area, which can assist in reducing pain and inflammation [23,24,25,26].

Furthermore, they aid in controlling and preventing hemorrhaging, correcting deformities, and maintaining pressure in cases where vein-related injuries are present. Using bandages is an effective and convenient wound care method that significantly facilitates the healing process [27]. The healing process of wounds can be complicated and often requires proper wound dressings that promote healing and prevent infection [22,27,28]. This review article aims to provide an updated overview of the different types of nanomaterials used in wound dressing, including nanofibers, nanocomposites, silver-based nanoparticles, lipid nanoparticles, and polymeric nanoparticles [29,30,31]. Additionally, this review highlights clinical studies of nanoformulations in wound healing to provide a comprehensive understanding of the efficacy and safety of these advanced dressings [32,33].

While several existing reviews have focused on the physical and biological properties, forms, advantages and disadvantages, and indications and contraindications of using nanomaterials in wound dressings, there is a lack of reviews that emphasize the wound healing process and the diverse types of nanomaterials used in promoting it [10,17,19]. Our review article aims to fill this gap by providing a comprehensive overview of the latest developments and applications of nanomaterial-impregnated wound dressings for wound care. Our review is distinctive in that it comprehensively covers a diverse range of nanomaterials, making our study highly relevant and up to date with the current research trends in the field of wound care using nanomaterials. Out of the 160 articles we referred to in this review, 53% were published within the past five years, further highlighting the timeliness and importance of our review article. Our review article also highlights the need for future advanced-design trials and the lack of high-quality evidence in the field. This is a crucial aspect to consider, as the ultimate goal of any wound dressing is to promote healing and improve patient outcomes. Moreover, our review is unique in that it also covers a wide range of clinical studies that have been conducted to evaluate the efficacy of nanomaterial-impregnated wound dressings in wound healing. Overall, this review article provides valuable insights into the individual nanoformulations used in wound dressings and their potential role in promoting wound healing and preventing infections.

## 2. Search Strategy

We conducted a comprehensive search of four databases, namely Scopus, Web of Science, PubMed, and Google Scholar, using the keywords “Dressing”, “Wound”, “Nanomaterial”, and “Nanoparticle”. Our search yielded a total of 730 research articles from all of the databases.

Inclusion criteria.

1. Articles that report original research and reviews on using nanomaterial-based dressings for wound healing
2. Articles that investigate the use of nanomaterial-based dressings for wound healing in in vitro, in vivo, or clinical studies
3. Articles that report on the synthesis, characterization, or modification of nanomaterials for wound healing applications
4. Articles published between 2001 to 2023

Exclusion criteria.

1. Articles that are not peer reviewed (e.g., case reports, commentaries, conference proceedings, abstracts, editorials)
2. Articles that are not written in English
3. Articles that are not published in peer-reviewed journals
4. Articles that report on the use of nanomaterials for other applications unrelated to wound healing
5. Articles that are not available in full text

**Figure 1:**
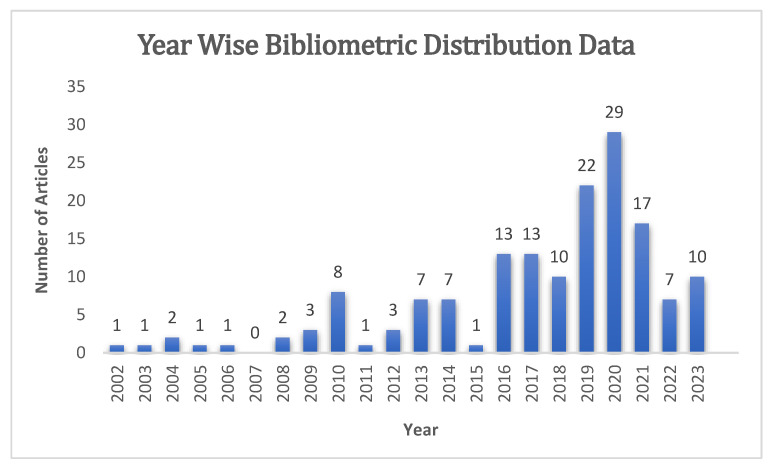
Year-wise bibliometric distribution data.

After applying inclusion and exclusion criteria, 160 articles were selected for review in this article. We also received some bibliometric data from a consulted bibliometrician, who provided information on the number of articles found by year. The articles selected for review were published between 2001 and 2023, with a year-wise distribution as represented in Figure 1.

Overall, our search strategy enabled us to obtain a diverse and representative sample of research articles in the field of wound healing using nanomaterial-based dressings.

## 3. Wound Healing Process

**Figure 2:**
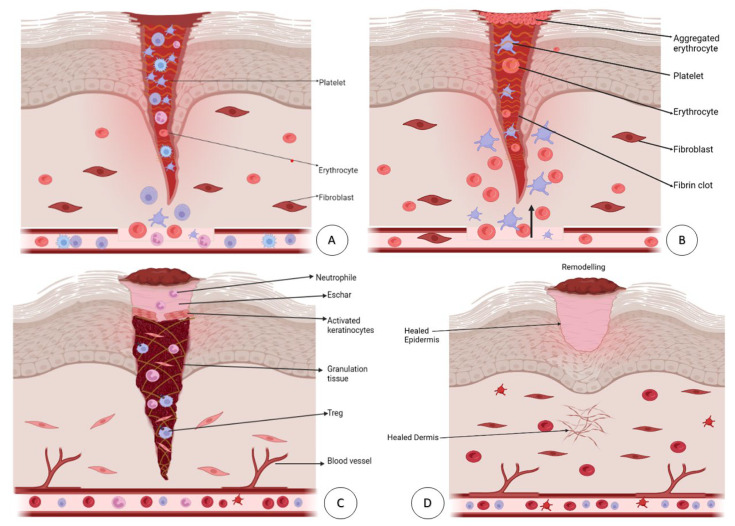
Normal wound healing process [36]. (**A**) Hemostasis—platelet aggregation and clotting cascade. (**B**) Inflammation—Inflammatory responses and autolysis by macrophages, neutrophils, and mast cells. (**C**) Proliferation—Fibroblast formation, epithelial cell regeneration, granulation, contraction, and angiogenesis. (**D**) Remodeling—Collagen remodeling from Type-III to Type-I.

The skin is an extensive and vital organ that serves various functions, the most crucial being protection against microorganisms. Any injury or damage to the skin increases the risk of infection, which can result in severe complications such as sepsis. The wound healing process involves four distinct phases: hemostasis, inflammation, proliferation, and tissue remodeling [2,34]. Each phase has been diagrammatically represented in Figure 2. These phases involve various cellular and molecular components, including platelets, macrophages, cytokines, growth factors, keratinocytes, fibroblasts, and other bioactive molecules, and interactions with extracellular matrix (ECM) components via integrin receptors. The mechanism of the hemostasis phase is initiated promptly following an injury to prevent excessive bleeding. However, this can lead to hypoxia and acidosis during the initial phase of wound healing [19,34].

Consequently, there is a reduction in vasoconstriction and an increase in the permeability of inflammatory cells. Platelets play a crucial role in blood clotting and produce a range of remodeling molecules, such as epidermal growth factor (EGF), platelet-derived growth factor (PDGF), histamine, serotonin, fibrinogen, and von Willebrand (VW) factors [35]. PDGFs, secreted from platelet granules, stimulate the mitogenicity and chemotaxis of neutrophils, macrophages, and fibroblasts at the injury site [36].

Following the initial hemostasis phase, the inflammatory stage of wound healing begins, characterized by the infiltration of various immune cells, such as neutrophils, monocytes, T cells, and mast cells, along with other mediators [2]. These mediators regulate the various aspects of wound healing, including inflammation control, angiogenesis, collagen accumulation, and epithelialization. Transforming growth factor β (TGF-β) and PDGF, which are involved in wound healing, play a significant role in the chemoattraction of immune cells [37,38]. In the proliferation phase, fibroblasts migrate to the wound site in response to chemotactic gradients of EGF, PDGF, and TGF-β. Fibroblasts are vital in initiating angiogenesis, collagen fiber formation, and epithelialization. The angiogenesis process is necessary for maintaining granulation tissue and is regulated by various molecules such as vascular endothelial growth factor (VEGF), TGF-β, and tumor necrosis factor-α (TNF-α) [39]. Matrix metalloproteinases (MMPs) promote angiogenesis by releasing VEGF and stimulating endothelial cells’ migration, proliferation, and survival. During wound healing, granulation tissue formation, collagen deposition, and angiogenesis co-occur with wound contraction and epithelialization. The final stage of wound healing is the remodeling phase, where the balance between cell formation and degradation results in well-organized tissue [2]. Newly formed epithelial cells emerge from the dermal edges and hair follicles, gradually bringing the wound edges together with wound contraction. Despite complete wound closure, the tissue at the wound site only retains 70–80% of its original tensile strength and remains vulnerable to damage. Although there should be no exudate at this stage, the overlapping nature of the phases necessitates careful consideration. During the recovery phase, the wound contact layer should provide adequate protection to newly formed epithelial cells, promoting healing and reducing the risk of injury [2,26].

The wound-healing process can vary based on the type of wound and the body’s ability to repair itself. However, in some cases, the non-healing or chronic wounds can become stalled or delayed in one or more stages of healing [2]. These types of wounds can pose a severe threat to the healthcare system and patients, and risk factors for chronic wounds can include a variety of medical conditions such as diabetes, immunosuppression, and peripheral vascular disease [2,15]. The inflammation usually lasts up to seven days in a normal wound-healing process. However, chronic wounds require longer due to increased inflammation caused by ECM degradation products. Therefore, the primary goal of wound management is to accelerate the healing process, both for acute and chronic wounds [7]. Achieving this objective requires employing methods that promote faster wound healing by enhancing the wound’s cellular and molecular processes. This can be achieved through advanced dressings, growth factors, and other bioactive agents that stimulate cell proliferation, migration, ECM remodeling, and neovascularization [22]. Proper wound care, infection prevention, and pain management are also essential considerations in effective wound management. By employing evidence-based wound care practices, healthcare providers can improve wound healing outcomes and promote better patient outcomes [2,15,35].

## 4. Nanomaterials Used in Wound Dressing

Nanomaterials, which refer to materials with at least one dimension in the nanometer range, have emerged as a promising class of materials for wound healing applications. They possess unique physical, chemical, and biological properties that can be tailored to meet specific requirements for wound healing, such as sustained drug release, enhanced cellular and tissue penetration, antibacterial properties, and controlled mechanical properties. In wound healing, nanomaterials have shown the potential to promote cell proliferation, migration, angiogenesis, and extracellular matrix remodeling and prevent infections. This section will discuss various nanomaterials explored for wound healing applications, including nanofibers, nanocomposites, graphene-based nanomaterials, titanium dioxide-based nanomaterials, nanoscaffolds, silver-based nanoparticles, lipid nanoparticles, and polymeric nanoparticles. We will provide a brief overview of the advantages and disadvantages of nanomaterials and their potential impact on wound healing.

### 4.1. Nanofibers

Nanofibers have garnered significant attention in the realm of nanotechnology and nanostructured materials over the past decade [40]. Nanofibers refer to fibers smaller than 100 nm or less than 1000 nanometers (sub-micrometer) produced through ultra-fine fiber production techniques such as electrospinning, which is widely used compared to other fabrication methods [41]. The electrospinning technique allows for the production of nanofibers with a higher surface area and dissolution rate, which can improve drug release [42]. The larger surface area to volume ratio of electrospun nanofibers enables them to load a large amount of drug, making them ideal for drug delivery applications [43,44,45].

Nanofibers also offer numerous advantages for wound dressing applications.

- Hemostasis: Nanofibers impart small interstices to the dressing and promote hemostasis due to their high surface area [46]. The physical feature of the nanofibrous dressings triggers the hemostasis mechanism without using a hemostatic agent [47].
- Absorptivity: Due to the high volume:surface area property of nanofibers, they absorb a high amount of water, ranging from 17.9–21.3%, whereas standard dressings only absorb 2.3% [41].
- Semi-permeability: Nanofibers have pores that are ideal for cell respiration and prevent wound desiccation. The semi-permeability of nanofibers regulates the moist wound environment and prevents infection. Furthermore, nanofibers promote gas permeation into and out of cells [48].
- Conformability: One of the criteria that must be tested is conformability or the ability to adapt to the shape of the wound. In the textile industry, it is widely accepted that the fineness of the fabric is related to its conformability. Finer fiber fabrics provide uniformity and covering to the wound, protecting it from infection [41].
- Functionality: Polymer nanofibrous membranes, which can be bioactive through electrospinning, can improve their application effectiveness. Electrospun nanofibers are multifunctional, bioactive dressings and can incorporate many therapeutic compounds [42,49].

#### 4.1.1. Nanofibers in Wound Healing

##### Polymeric Nanofibers

The selection of polymers for fabricating nanofibers is crucial for wound care, as it influences the resulting dressing’s mechanical strength and therapeutic activity. Natural and synthetic polymers are both viable options, offering unique advantages and disadvantages [22]. Different polymers with different molecules and origins can result in differences in properties such as the spinning solution’s viscosity, nanofibers’ morphology, biocompatibility, and mechanical and physicochemical properties [50].

Chitosan is a natural biodegradable polysaccharide derived from chitin deacetylation. It has the same glycosaminoglycan component as ECM, apart from the ability to be biocompatible, biodegradable; it also has hemostatic, antibacterial and antifungal properties which is unique to chitosan [51,52]. This advantage provides faster diabetic foot ulcer recovery. However, chitosan’s poor mechanical strength can limit its use, so it is often combined with other polymers such as collagen or gelatin to strengthen its mechanical properties [53]. Collagen is a vital constituent present in ECM. Collagen stimulates fibroblast migration in the damaged tissue and modulates the granulation process, helping in cell attachment and proliferation [51]. However, it is commonly combined with synthetic polymers because it is unstable at higher temperatures [54]. In contrast, synthetic polymers are preferred in nanoparticle preparations due to their flexibility, stiffness, and superior mechanical strength [50].

Poly(lactic-co-glycolic acid) and polycaprolactone are two biodegradable synthetic polymers often used for manufacturing nanofibers due to their stability and compatibility with the extracellular matrix [55,56]. PCL imitates the ECM; its cell attachment and proliferation ability has been restricted for hydrophobic properties. Since PCL has the main disadvantage of high porosity, it can be resolved by co-using with natural polymers such as collagen, gelatin, and chitosan, thus becoming a good choice for fabrication [50]. Polyvinyl alcohol and poly(ethylene oxide) are other synthetic polymers with good mechanical properties and resistance to chemical agents, making them viable choices for antibacterial wound dressing. Polyvinyl alcohol (PVA) is soluble in water; it is non-toxic and noncarcinogenic [57]. PVA with pluronic and polyethyleneimine (PEI) is a good choice in antibacterial wound dressing [40,58]. Additionally, silver with PVA is considered a good choice in antibacterial wound healing. Poly (ethylene oxide) (PEO) is a well-known hydrophilic carrier for nanoscale active ingredients [50]. PEO offers a desirable healing environment for the DFU. PEO has shown benefits in producing long, bead-free fibers when combined with cyclodextrin and chitosan. Herbal drugs are increasingly incorporated into electrospun nanofibrous mats to develop safe and effective wound dressings. One study sought to investigate the use of herbal drugs in wound dressings by incorporating thymol into electrospun nanofibrous mats made of poly(e-caprolactone) (PCL), poly (lactic acid) (PLA), and their 50/50 hybrid. Results showed that the hybrid nanofibrous mats containing thymol were effective against *Staphylococcus aureus*. Additionally, in vivo animal studies were performed on wounded rats and observed a 92.5% wound-closure percentage after a 14-day treatment period [56]. These findings imply that incorporating herbal drugs into electrospun nanofibrous mats may be a promising approach to developing safe and effective wound dressings [59,60].

Curcuma longa has a wide range of medicinal applications as an anti-inflammatory agent. It has chemopreventive properties and antioxidant, anti-amyloid, anti-arthritic, anti-HIV, antimicrobial, and thrombo-suppressive properties [61,62]. Curcumin’s most effective action is in scavenging reactive free radicals of oxygen and nitrogen. The nanofibers are said to have an optimal structure for wound healing and, when combined with curcumin, can be made into a highly efficient wound dressing because of their anti-inflammatory and antioxidant properties [63]. Appropriate solvent use during the preparation of curcumin nanofibers and controlled release of curcumin is necessary to achieve improved bioavailability [64]. Merrell et al. [63] developed curcumin-loaded PCL nanofibers for diabetic wound care. Curcumin promotes the migration of various cells in the wound bed, including myofibroblasts, fibroblasts, endothelial cells, and macrophages, accelerates epidermal re-epithelialization, and enhances neovascularization and collagen deposition at lower concentrations [52,65].

##### Copper Nanofibers

Copper plays a critical role in skin formation by aiding in synthesizing and stabilizing extracellular matrix proteins and promoting angiogenesis. Furthermore, copper’s broad-spectrum antibacterial efficacy further highlights its importance in skin health [66]. Copper oxide-impregnated wound dressings were recently clinically approved for treating acute and chronic wounds [67]. A study was conducted to investigate the effectiveness of the dressings in patients with different medical conditions. Ten cases were presented in which copper oxide-impregnated wound dressings facilitated wound healing by eliminating infections, reducing necrotic tissue, and promoting wound closure [21,66]. The findings are consistent with in vitro and animal studies demonstrating copper’s role in skin regeneration and angiogenesis. Copper oxide-impregnated wound dressings could be an effective treatment for difficult-to-heal wounds [67].

Anqi Zhan and his coworkers designed a multifunctional nanofiber loaded with bioactive agents (chitosan (CTS), copper (Cu), and decellularized Wharton’s jelly matrix (DWJM)). The nanofiber was designed to release chitosan and copper in a controlled manner to induce antibacterial and angiogenic responses, respectively. DWJM was also incorporated to enhance collagen deposition. In vitro and in vivo analyses demonstrated the efficacy of this approach, with the sequential release of bioactive agents promoting antibacterial activity, cellular migration, neovascularization, and collagen deposition. The platform demonstrated satisfactory mechanical properties, absorption, biodegradability, biocompatibility, and adhesive rates. Overall, the core-shell CTS-PCL Poly(L-Lactide-co-caprolactone)/DWJM-Cu nanofiber platform holds great promise for treating infected diabetic wounds [68]. Another research study developed a nanofibrous electrospun mesh composed of avocado extract, copper nanoparticles (CUPs), and polyurethane, showing positive results in wound healing. The selective albumin adsorption over fibrinogen was observed by protein adsorption experimental analysis [69]. The composite scaffold exhibited excellent physicochemical properties and selective protein adsorption, making it suitable for wound healing applications. The synthesized mesh also provided a high hemocompatibility surface that delayed blood coagulation and decreased red blood cell damage [70]. Usually, CUPs are said to have antimicrobial activity and are often used in wound healing, skin remodulation, and anti-inflammatory therapy, which also benefits the formulation. Copper is a cofactor for enzymes such as superoxide dismutase and cytochrome oxidase. Copper promotes angiogenesis in wounds by inducing the expression of endothelial vascular growth factors. These factors promote wound healing by improving angiogenesis, reducing inflammation, and boosting immunity. Therefore, biosynthesized CUP-decorated nanofibrous polyurethane mesh loaded with avocado extract can promote successful wound healing, especially in chronic wounds, by protecting against microbial attacks and enhancing the process of regeneration [38]. Further, a research study was conducted to assess the effect of copper nanoparticles on anti-biofilm activity. Hence, copper particles were incorporated into nanofibers during the electrospinning of poly-D, L-lactide (PDLLA), and poly(ethylene oxide) (PEO), resulting in nanofibers containing copper particles (Cu-F). The ability of the Cu-F nanofibers to prevent biofilm formation was tested using *Pseudomonas aeruginosa* PA01 and *Staphylococcus aureus* Xen 30. After 48 h, the Cu-F nanofibers reduced biofilm formation by 41% and 50% for *P. aeruginosa* PA01 and *S. aureus* Xen 30, respectively. The observed reduction in biofilm formation was attributed to the copper released from the nanofibers. These findings suggest that copper-containing nanofibers may be a promising component in developing wound dressings. The antimicrobial properties of copper and its ability to prevent biofilm formation make it a potential solution to combat infections associated with wound healing [66].

### 4.2. Nanocomposites

Nanocomposites are composites with at least one component measured in nanometers. Nanocomposite materials have emerged as a viable substitute for overcoming the limitations of monolithic and micro composites, despite the challenges associated with regulating composition and stoichiometry [71,72]. Nanocomposites are materials that have nanometer-sized particles mixed in with a polymer matrix. Due to their small size, the dispersion of the nanocomposite filler is much better than in conventional composites or pure polymers [72]. This improved dispersion results in significantly enhanced material properties, including increased modulus and strength, exceptional barrier properties, and resistance to high temperatures and solvents. Furthermore, nanocomposites are less flammable than conventional materials [73]. Because of these advantages, nanocomposites are a promising alternative to conventionally filled polymers. Nanoparticles possess composite-like properties due to their small size. The small inter-particle distances of nanoparticles lead to a significant fraction of the polymer matrix near their surfaces being converted into an interphase of different properties [74]. The morphology of nanoparticles can also contribute to their composite-like properties. Overall, the composite-like properties of nanoparticles make them useful in various applications [73]. Integrating nanocomposite properties, such as strength, hardness, toughness, and stability enhancements, and incorporating bioactive properties with mechanical properties will help produce a new generation of medical devices, implants, and prosthetics [74]. Nanocomposites offer unique opportunities to overcome barriers in the medical, food packaging, pharmaceutical, electronics, and energy sectors. Polymer nanocomposites have amazed all polymer industries because of their unique ocular, electric, catalytic, and biomedical properties [58].

#### 4.2.1. Classification of Nanocomposite Materials

Nanocomposite materials can be classified into polymer matrix nanocomposites, ceramic matrix nanocomposites, and metal matrix nanocomposites [75].

Polymer matrix nanocomposites (PMNC) are commonly referred to as fillers. These fillers are grouped into linear (1D, e.g., nanotubes of carbon), layered (2D, e.g., montmorillonite), and powder (3D, e.g., silver nanoparticles (AgNPs)) forms. PMNCs have high thermal stability, excellent mechanical properties, and lower gas permeability (higher barrier efficiency) [41]. One or more composites are intentionally added to ceramic matrix nanocomposites (CMNs) to improve wear resistance and thermal and chemical stability. The disadvantage of ceramics in industrial applications is its low toughness and brittleness. An example of a CMN includes a matrix in which energy-dissipating constituents (fiber or particles) are integrated to minimize fragility and increase fracture durability. Metal matrix nanocomposites (MMNCs) are multiphase materials consisting of alloy matrix or ductile metal in which some nanosized materials are inserted for reinforcement. MMNCs exhibit high ductility, resilience, strength, and modulus [74].

Advantages of nanocomposites [75].

Nanocomposites have the following advantages over other composite materials.

- High surface/volume ratio allows for small filler size and inter-fill distance.
- Improved mechanical properties, high strength.
- Resistance to scratches.

Disadvantages of nanocomposites.

- Impact efficiency correlated with the incorporation of nanoparticles into a composite bulk matrix.
- Insufficient understanding of formulation properties.
- Structural relationship, need for easier exfoliation of particles, and dispersion.
- Cost-efficiency.

#### 4.2.2. Nanocomposites in Wound Healing

**Figure 3:**
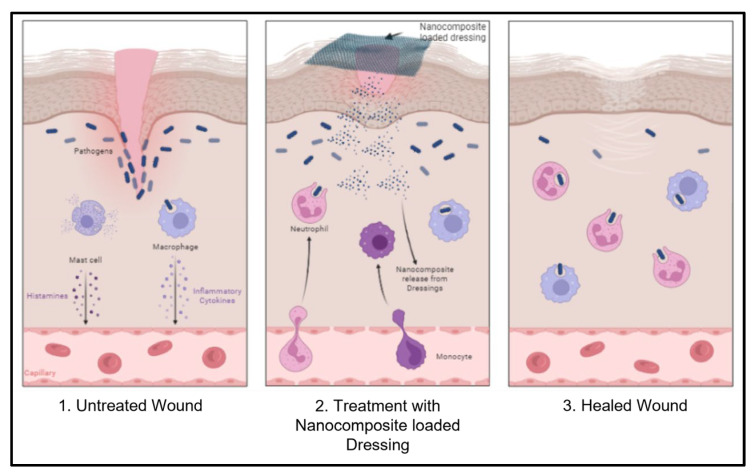
Nanocomposite-impregnated dressing for wound treatment; (**1**) Inflammatory response: skin wound. (**2**) Wound treated with nanocomposite loaded dressing. (**3**) Healed wound.

Nanocomposite dressings have significant antibacterial effects and good biocompatibility with the fibroblast cells NIH3T3 and L929. Nanocomposite dressings exhibit synergistic effects attributed to their combination of properties, such as potent antimicrobial action, excessive swelling, high moisture-vapor transmission rate, good hydrophilicity, biocompatibility, and promotion of wound closure. The presence of nanoparticles, which can disrupt bacterial cell membranes and inhibit their growth, contributes to the antimicrobial activity of nanocomposite dressings [76]. The mechanism of biological activity of nanocomposites is demonstrated in Figure 3. Furthermore, the excessive swelling properties of nanocomposite dressings aid in maintaining a moist wound environment, which is critical for wound healing. Moreover, nanocomposite dressings’ high moisture-vapor transmission rate allows for exchanging oxygen and other nutrients between the wound and the surrounding environment. The hydrophilic nature of nanocomposite dressings also contributes to this exchange by allowing easy absorption of wound exudate [38,40].

Antibiotic misuse and the incidence of bacterial infections in wounds have become serious issues for patients and medical systems. Researchers have developed a conductive, self-healing nanocomposite hydrogel with photothermal antibacterial properties to treat infected wounds. These hydrogels, based on N-carboxyethyl chitosan (CEC) and benzaldehyde-terminated Pluronic F127/carbon nanotubes (PF127/CNT), demonstrate a suitable gelation time, stable mechanical properties, hemostatic properties, high water absorbency, and good biodegradability. In vitro and in vivo experiments indicate that hydrogel can improve wound closure healing, collagen deposition, and angiogenesis. The findings suggest that these hydrogels have great potential as a multifunctional wound dressing for treating infected wounds [77].

Wound infections caused by multidrug-resistant bacteria are a global health threat. The rising incidence of microorganisms resistant to several antibiotics complicates wound infection management. To address this problem, researchers have developed wound dressings using chitosan, a biocompatible material that stabilizes 2-mercapto-1-methylimidazole-capped gold nanocomposites (CS-Au-MMT). The unique combination of AuNPs, MMT, and CS in these dressings has been found to disrupt bacterial membranes effectively. Tests on cell-based wound infection models and in vivo rabbit wound healing models have also shown that the CS-Au-MMT/gelatin dressing is biocompatible and has significant potential to treat methicillin-resistant *Staphylococcus aureus*-associated wound infections. According to these findings, the CS-Au-MMT/gelatin dressing is a promising candidate for future biomedical applications in wound care [78]. Another research study demonstrated a novel approach involving the use of bacterial cellulose (BC) decorated with 4,6-diamino-2-pyrimidinethiol (DAPT)-modified gold nanoparticles (Au-DAPT NPs) as a wound dressing (BC-Au-DAPT nanocomposites). Compared to conventional antibiotics, these nanocomposites have demonstrated greater efficacy against Gram-negative bacteria, such as *Escherichia coli* and *Pseudomonas aeruginosa*. In rat models with full-thickness skin wounds infected with *E. coli* or *P. aeruginosa*, BC-Au-DAPT nanocomposites successfully inhibited bacterial growth and promoted wound repair. Hence, the BC-Au-DAPT nanocomposite system demonstrates promising results for treating infected wounds caused by multidrug-resistant bacteria [79].

Furthermore, nanocomposite dressings are biocompatible, so they do not react negatively when in contact with living tissue. Nanocomposite dressings’ ability to promote wound closure and improve wound appearance is also due to their composite-like properties, which increase mechanical strength and flexibility. Combined with these properties, nanocomposite dressings are an excellent choice for wound healing applications [38].

##### Bacterial Cellulose Nanocomposite

Bacterial cellulose is an important biomaterial used in various fields due to its unique properties, such as mechanical strength, purity, degree of polymerization, and water-holding capacity [80]. Bacterial cellulose is a fibrous composition of a 3D structure, a non-woven network of microfibrils with an analogous chemical structure of plant cellulose, which is linked by inter and intra-fibrillary H-bonding, resulting in bacterial cellulose in a non-dried state or high-strength hydrogel [81]. It has characteristics such as up to 99% high purity water content, polymerization degrees, crystallinity (around 70–80%), and mechanical stability. It is widely used in wound dressings, artificial skins, blood vessels, and biomembranes [82]. It can be an ideal wound dressing material since it controls the exudation of the wound and provides a moist environment leading to better wound healing [83]. Because bacterial cellulose has no antimicrobial activity in preventing wound infection, much effort has been expended in developing antimicrobial silver particles containing bacterial cellulose membranes. Isolating bacterial cellulose, on the other hand, is time-consuming because it is challenging to sustain bacterial fermentation cultivation for several days. Bacterial strains can lose their ability to synthesize cellulose and thus lose their integrity [84,85].

### 4.3. Graphene-Based Nanomaterials

Inorganic nanoparticles have displayed vital capabilities for enhancing the mechanical properties of matrices of the polymer [86]. Numerous nanoparticles, such as calcium phosphate, hydroxyapatite, and natural clays, can be used as fillers to augment characters [87]. Graphene is an allotropic form of carbon with densely packed planar sheets of carbon atoms within the crystal lattice. It has emerged as a highly promising nanomaterial with numerous applications in biomedicine since its discovery [88]. Because of their large surface area and chemical and physical properties, graphene-based nanoparticles are excellent candidates for use as nanoscale building blocks in biological research [89]. Strong van der Waals attractions between the molecules are very helpful in designing nanocomposites [18].

#### 4.3.1. Graphene Oxide

Graphene oxide (GO) is a highly oxidized graphene derivative with intriguing biocompatibility. Incorporating GO into a polymer matrix can improve cell–scaffold interactions and nanocomposite mechanical properties [88]. Graphene oxide has been substantially explored as a better reinforcement for biopolymers such as cellulose and chitosan. Graphene oxide shows high drug-loading capacity because of its large surface area. Zein (a corn protein) is a versatile material used in wound dressings, tissue engineering, scaffolds, and drug delivery systems. The degradation products of zein can aid in cell proliferation. Graphene oxide and zein have demonstrated excellent wound-healing effects by improving the wound-healing process via their bactericidal properties [87]. In graphene-based composites such as fillers, graphene is utilized as a base for the dispersion or incorporation of other nanomaterials, polymers, and metal nanostructures [89]. These composites are prepared mainly by chemical reduction, microwave-assisted growth, solution mixing, and hydrothermal methods. Graphene can also be incorporated into dressings along with silver oxide (AgO), zinc oxide (ZnO), and copper oxide (CuO), or as Ag-graphene polymer hydrogel for rapid wound healing [18,87]

Secondary infections can occur as a result of microbial attack after skin injury. Commercially available wound dressings are required in the early stages of injury, but they have the disadvantage of quickly adhering to the wound surface and causing trauma to the healing injury. Thus, graphene-based nanoparticles in the form of fibers or hydrogel can increase the wound healing process by overcoming this drawback and also due to their high antibacterial action [86]. Covalent techniques are being used to combine different polymers with graphene molecules to synthesize hybrid materials with desired properties. Chitosan is a non-toxic, biodegradable, biocompatible, natural polymer with applications in tissue repair, wound repair, antimicrobial activity, and cell adhesion. Recently, a few reports were presented on chitosan/GO hybrid systems with enhanced mechanical properties [90,91]

#### 4.3.2. Graphene Preparations and Their Antibacterial Spectrum

Lu et al., 2019, have prepared graphene oxide-based antibacterial cotton fabrics using three different techniques: direct absorption, chemical crosslinking, and radiation-induced crosslinking. These fabrics have high antibacterial activity (bactericidal in nature—98%), can maintain a high inactivation efficacy even after washing, and are non-irritant on the wound surface [92]. Karimi et al. developed self-cleaning nanocomposite-coated fibers containing graphene-titanium dioxide (TiO_2_) [93]. These fabrics have been found to have excellent antibacterial activity against *Staphylococcus aureus* and *Escherichia coli* and antifungal activity against *Candida albicana*, with no cytotoxic effects. Ag-graphene composites containing N, N′-methylene bisacrylamide and acrylic acid with different mass ratios were prepared. At a mass ratio of 5:1, the silver-graphene composite hydrogel showed the highest swelling percentage. The best part is that it has the potential to accelerate healing and completely reconstruct the epidermis [94]. Graphene is also a good candidate for drug delivery due to its large area, surface chemistry, high efficiency loading capacity, and specific geometry [88].

### 4.4. Titanium Dioxide-Based Nanomaterials

Titanium dioxide (TiO_2_) is a multipurpose biomaterial with bioactive and bacteriostatic properties [95]. TiO_2_ is considered an antimicrobial agent due to its broad-spectrum antibacterial potency, protection, and stability. Previous research suggested that medicinal plants, engineered metal oxide, and metal nanoparticles were more environmentally friendly and cost-effective than chemically and physically engineered nanoparticles [30,94]. Nano-TiO_2_ is said to have regulated the healing process by increasing skin moisture (hydrophilic activity) and antimicrobial properties [96]. TiO_2_ nanoparticles occupy a unique position due to their high corrosion resistance and favorable biological effects. TiO_2_ nanotube films have been extensively studied as growth support and adhesive platforms for stem cells and to prevent bacterial adhesion and blood coagulation for hemorrhage control [97,98]. TiO_2_ also acts as an excellent antimicrobial agent when combined with silver. TiO_2_ is considered non-toxic, and the American Food and Drug Administration (FDA) has approved its use in medications, human food, food contact materials, and cosmetics [99].

The potential of TiO_2_ to photocatalyze the developed reactive oxygen species (ROS) products is further improved when the particles are ground to nanometer dimensions, thus increasing the electron flux and surface area. For instance, it has been demonstrated that they can trigger inflammatory reactions in humans and animals when ground into particles smaller than 20 nm. The study was conducted without light, and the results revealed that the penetration and attachment of nanoparticles could be the reason why cells generate oxidative stress and trigger the production of ROS [100]. Particles coated with a polymer brush did not bind to the cell membranes and, therefore, did not enter the cells, reducing the production of reactive oxygen species and thus enabling normal cell functioning [101]. Alteration of mesoporous silica nanotubes with nanocrystalline TiO_2_ (anatase) led to the development of photoactivated antibacterial agents. The analysis of the TiO_2_ antibacterial and photocatalytic performance showed that nanotubes have low toxicity [102]. The TiO_2_ nanoparticle-reinforced zein–PDA polymeric nanofibrous scaffold offers excellent thermal stability. The fibrous network is highly interconnected and mimics natural ECM, promoting cell proliferation and adhesion. The significance of the zein–PDA –TiO_2_ scaffold’s nano topographic structure to cell movement has demonstrated the scaffold’s suitability for wound healing applications [97,98,103].

TiO_2_ nanoparticles/gelatin composite offer possible benefits in improving and accelerating wound healing through the proliferation of fibroblasts. Further, TiO_2_ nanoparticles/gelatin composite accelerate the wound repair and stabilize the damaged area by reorganizing granulated collagen fibers and tissue. Additionally, the antibacterial activity of TiO_2_ nanoparticles enhances wound healing by preventing the hydrophilic effect of gelatin and wound infection, preserving wound moisture [104]. A newly developed dressing containing synthetic polymer (N-vinylpyrrolidone), chitosan biopolymer, and TiO_2_ nanoparticles had a strong antibacterial effect on four pathogenic bacteria and was non-toxic to L929 and NIH3T3 fibroblasts. The closure rate of the nanocomposite-treated wounds was successful in an albino rat full-thickness wound model compared to that in the negative control groups. *Moringa oleifera* (*Moringaceae* family) aqueous leaf extract was utilized by V. Sivaranjani et al., 2015, using a low-cost, time-saving, simple, environmentally friendly synthetic method to synthesize TiO_2_ nanoparticles that also displayed important wound-healing behavior in albino rats [105,106]. For the synthesis of nanoparticles using leaf extract, an eco-friendly green chemistry method will increase sustainable management and economic viability.

Electrospun TiO_2_ nanofiber was developed for wound dressing with polyethyleneimine, pluronic F127, and polyvinyl alcohol (PVA) mixed solution with enhanced antibacterial activity. PVA-Plur-PEI/TiO_2_ nanofiber may be a promising method for improving wound care, skin infection treatment, and tissue regeneration [95]. Moreover, it has been stated that TiO_2_ triggered by ultrasound can also accelerate the healing process in mouse wounds infected with *E. coli.* The ultrasound-irradiated TiO_2_ (UIT) produces ROS, which mediate a positive cellular response at physiological levels, such as in angiogenesis. It should, however, be noted that excessive production of ROS can cause cell damage. Precautionary measures must, therefore, be applied while using UIT. Given that the benefits of UIT outweigh its disadvantages, its applicability as a novel therapeutic wound healer may be well-accepted and appreciated. Because of their extensive use, the possible biological effects of TiO_2_ nanoparticles have become increasingly significant, and concerns about health risks to the general population and exposed workers are increasing. In contrast, however, some previous studies have suggested the non-toxic nature of TiO_2_ nanoparticles [107].

### 4.5. Nanoscaffolds

Nanomaterials are efficiently used to develop nano polymeric scaffolds with fibrous properties and characteristics of ECM nanoscale [108]. Self-assembly, phase separation, and electrospinning are the various methods used to build nanomaterial-based scaffolds [109]. These techniques successfully develop polymeric nanofibers with pores, which generate fibroblasts in wounds and show structural and physical characteristics identical to those of ECM. Nano-fibrous scaffolds have a high surface-to-volume ratio that is expected to improve cell adhesion. Several factors must be considered when developing scaffolds for tissue engineering applications. Tissue engineering is an integrative field that uses engineering and life science principles to develop biological substitutes that restore, maintain, or improve tissue function. Biomaterials play a crucial role in tissue engineering by helping as three-dimensional matrixes for proliferation, cellular growth, and new tissue formation [110].

Shahverdi et al. [55] effectively utilized silk fibroin/PLGA as hybrid scaffolds by cultivating fibroblast cells to promote diabetic wound healing. In another research study, electrospun scaffolds blended with nanofibrous chitosan polyvinyl alcohol were effectively used for treating rats with diabetic wounds, with progressive rates of healing in contrast to controls. Using in vivo examination in Wistar rats, a study demonstrated the potency of electrospun nanofiber membrane with AgNPs with decreased inflammation, enhanced wound healing, long-lasting antibacterial action, and low cytotoxicity. Fu et al. demonstrated rapid wound healing using electrospun curcumin-loaded poly (-caprolactone)-poly(ethylene glycol)-poly(-caprolactone) fibrous mats. The composite electrospun scaffold showed antioxidant effects and low cytotoxicity in vitro and improved the healing of wounds in vivo [64,111].

Injectable scaffolds must have mechanical strength comparable to that of the defective site to ensure structural stability and facilitate mechanical transduction [112]. Biomaterials such as synthetic and natural polymers, bioceramics, and biocomposites were used to synthesize injectable scaffolds such as hydrogel, microparticles, nanoparticles, and nanocomposite films. Hydroxyapatite and chitosan, among other biomaterials, have been extensively studied for injectable scaffold fabrication. A study was conducted on microparticles by adding porosity and integrating nano-hydroxyapatite (nHA) into spherical chitosan scaffolds. Integrating nHA into chitosan amplified the scaffolds’ maximum compressive strength. The study also found that the mechanical strength of scaffolds could be further enhanced by using a simple treatment to reduce scaffold pore sizes. Increased nHA promotes proliferation and adhesion to scaffolds [113]. According to research, the MFC/NFC method produces strong scaffolds for musculoskeletal purposes such as fractures, rotator cuff injuries, and the nonunion of skeletal bone [83]. The MFC/NFC technique has yielded organic solvent-free, synthetic scaffolds showing cytocompatibility that will assist mechanical support during healing.

The combination of sophisticated scaffolding manufacturing technologies, such as solid freeform manufacturing (SFF) and thermally induced phase separation (TIPS), has produced complex scaffolds with regulated structural features in various sizes. When attempting to improve the disadvantages of both approaches, this approach takes advantage of their complementary benefits. TIPS offers the potential to create a nano-fibrous pore wall architecture that has been proven to function over more conventional smooth-walled scaffolds and enhance cell behavior, whereas SFF allows for easy control of the internal and external scaffold structure. These two methods are suitable for several types of scaffolding improvements; therefore, scaffolds constructed with SFF and TIPS can be tailored to meet the needs of various tissue engineering applications. According to one study, the copper-assisted hydrothermal deposition method provides an easy and manageable way of producing a nano/micro-structured surface on hydroxyapatite scaffolds that benefits wound recovery and angiogenesis [113]. Surface-modified magnetite (Fe_3_O_4_C_16_) nanoparticles functionalized with limonene and eugenol exhibit anti-adhesive and antibacterial properties, which are critical for wound regeneration functions. Similarly, gentamicin-filled zinc oxide nanoparticles combined with a three-component chitosan gel exhibited synergistic antibacterial action with greater growth-inhibiting potential against *P. aeruginosa* and *S. aureus* than gentamicin alone; thus, this finding suggested that such a nanocomposite could be used at the wound site for wound healing and prevention of infection. In another study, AgNPs were dispersed in both in vitro and in vivo wound models in a polyethylene cloth [17]. Scaffolds crosslinked with varying concentrations of AgNPs and two different types of nano-HAp were characterized for surface microstructure, thermal properties, cell proliferation, and cell viability. Adding gold nanoparticles and nano-HAp to scaffolds was hypothesized to increase cell proliferation and viability compared to non-nanoparticulated scaffolds [114].

Ghosh Auddy et al. [94] attempted a study in which a new cationic biopolymer guar gum alkylamine containing AgNPs was developed to improve the healing of wounds. Preclinical analysis of fibrous mats/scaffolds for wound dressing material was performed utilizing electrospun material composed of chitosan oligosaccharides containing AgNPs (15–22 nm) and polyvinyl alcohol using the same combination strategy [17]. The material had outstanding antibacterial action against *S. aureus* and *E. coli* without revealing toxicity inside the wound site. Biomimetic scaffolds, which are three-dimensional with anisotropic and heterogeneous characteristics that resemble those of native heart valve tissue, were advantageous for the effective in vitro production of tissue-engineered heart valves (TEHV). Three-dimensional anisotropic composite scaffolds comprising methacrylate hyaluronic acid (Me-HA), polyacrylonitrile (PAN), and bioactive hydrogel nano-micro-fibrous woven fabric have been developed and characterized [76]. These composite scaffolds gave micro-environmental signs and adequate support for the proliferation and deposition of extracellular matrix (ECM) while maintaining the fibroblastic phenotype of normal human aortic valve interstitial cells (nHAVIC) and restricting the pathological differentiation of diseased HAVIC (DHAVIC) into osteoblasts and myofibroblasts compared to woven fibers or pure hydrogels [115]. The nanotopography of a wound-dressing scaffold is currently one of the primary goals of scientists to sustain a high rate of wound closure paired with decreased formation of a scar [116]. With this aspect, a study illustrated that the density of nano grooves and orientation during wound healing are critical for fibroblast migration and thus emphasized the importance of the scaffold’s nanotopography in wound healing [17].

### 4.6. Nanoparticles Used in Wound Healing

Nanoparticles have gained increasing attention as a promising strategy for improving wound healing outcomes [9]. Nanoparticles possess unique physical, chemical, and biological properties that make them attractive for wound healing applications [31]. Advanced nanoparticles can be engineered to have specific characteristics, such as the controlled release of bioactive agents, enhanced cellular and tissue penetration, and antibacterial properties [117]. Incorporating nanoparticles into wound dressings has shown great potential in promoting wound healing by stimulating cell proliferation, migration, and differentiation, accelerating angiogenesis and ECM remodeling, and preventing infection. Moreover, nanoparticle-based wound dressings have been shown to improve the mechanical properties of the dressing, increase its durability, and enable sustained drug release over an extended period [118]. This review will discuss the latest developments in advanced nanoparticle-based wound dressings, their properties, and their potential applications in wound healing.

#### 4.6.1. Silver-Based Nanoparticles

In the early 1970s, silver-based compounds (silver sulphadiazine) in wound care were introduced to provide broad-spectrum and inexpensive treatment. Silver-containing dressings can decrease the wound’s bioburden and time required for healing. Silver (Ag^+^) ion is the active antimicrobial factor that can cause reactive oxygen species production by interfering with the thiol group. Recently, AgNPs gained utmost attention in anti-inflammation and reduction of the wound bioburden since they can release Ag^+^ ions at a rate higher than bulk silver (because of their large surface area). They also can cross biological compartments if released from dressings. Silver is inert but contact with an aqueous environment can ionize it to Ag^+^, the active antimicrobial agent [119,120].

It has been shown that after absorption of Ag^+^ by *E. coli*, the ions inhibit phosphate uptake, a process reversible by the presence of thiols (–SH), indicating the role of protein thiol oxidation in toxicity. The dressings can be designed for sustained release into the wounds or by chemical attachment of Ag^+^ to the dressing material for antimicrobial effects on the exudate extracted from the wound [120,121]. Early signs of their efficacy were encouraging, and by similar mechanisms, many experiments have shown that nanosilver-impregnated dressings exhibit higher antimicrobial effects than the bulk silver-containing formulation [121].

##### Applications of AgNPs

A chitosan-poly ethylene glycol (PEG) hydrogel impregnated with AgNPs was developed to boost wound healing in diabetic patients. Chitosan-PEG solution containing silver nanoparticles was produced by reducing silver nitrate with PEG and chitosan solution. The resulting prepolymer solution was crosslinked to produce the desired hydrogel using glutaraldehyde. The hydrogel displayed a sustained release of Ag nanoparticles for a minimum of 7 days, exhibiting the slow biodegradation of the hydrogels. The improved antioxidant, anti-inflammatory, antimicrobial, and wound healing results demonstrated that the chitosan-PEG hydrogel associated with silver nanoparticles may be an excellent material for wound dressings in the case of chronic diabetic wounds. AgNPs can be of essential aid in preventing all forms of infections in chronic diabetic and non-healing wounds [122].

Chitosan is a chitin derivative obtained from the partial deacetylation of natural chitin. It is an excellent injury-healing substance because of its biodegradability profile, biocompatibility, cell binding capability, bactericidal activity, and wound-healing effects. A wound healing experiment showed an increase in the healing of diabetic wounds in rabbits using Ag nanoparticles and chitosan. Chitosan-PEG hydrogels with AgNPs added have proved to be special antibacterial agents because of their efficient electrical, thermal, and optical properties for wound dressings [122,123]. Chitosan influences every step of injury healing process, so it is a marvelous candidate for accelerating wound healing and an excellent material for increasing wound tensile strength. AgNP-integrated chitosan-PEG hydrogel was highly effective in promoting wound healing compared to plain chitosan hydrogel. The treatment group showed enhanced wound contraction, re-epithelialization, and keratinocyte migration, while the control group exhibited minimal granulation tissues and inflammatory infiltrations. The results indicate that the AgNP-integrated chitosan-PEG hydrogel could significantly improve the healing process of wounds. During a biological assessment, excellent antioxidant and antibacterial properties were observed with AgNP-loaded chitosan-PEG hydrogels, thus inspiring their use for the treatment of diabetic rabbit tissue injury. A comparison of in vivo experiments conducted using AgNP-impregnated chitosan-PEG-hydrogel and chitosan–PEG hydrogels showed that engineered medicines have better efficacy in wound treatment [122].

#### 4.6.2. Lipid Nanoparticles

Lipid nanoparticles (LNPs) are single- or double-layered nanosized natural or semisynthetic lipids used as drug carriers to improve bioavailability. LNPs have better skin adhesion properties, thus producing a sealing effect on the skin, moisture loss prevention, and enhanced drug absorption [31]. Thus, integrating LNPs into the wound dressing can be highly effective. Since these are skin-targeted deliveries, adverse effects are minimized [92]. They may be vesicular systems such as liposomes, niosomes, ethosomes, solid lipid nanoparticles, nanostructured lipid carriers, or micellar systems.

##### Solid Lipid Nanoparticles

Solid lipid nanoparticles (SLNPs) are submicron-sized dispersions (composed of a lipid core with surfactants/emulsifiers and a solvent system) [29]. Since they are both hydrophilic and lipophilic (because of surfactant action), they can be employed in delivering polar or nonpolar drugs [5].

- Curcumin and Ampicillin-Loaded SLNPs

Topical ampicillin could be used after a surgical procedure to minimize the chances of infection. Since curcumin is known for its natural antibacterial properties, it can be combined with ampicillin and incorporated into lipid nanoparticles [124]. The antimicrobial efficacy of curcumin- and ampicillin-loaded SLNPs shows synergistic effects in wound healing. Curcumin SLNPs can also be used along with silver hydrogel compositions to increase the antibacterial activity. Animal studies were conducted by applying the formulation to the burned skin of an animal and observing the wound-healing process for 14 days [125]. The results suggest that using nanoformulations of curcumin and ampicillin considerably boosts healing rates [125,126].

- Curcumin-Loaded Collagen NP Cryostructures

Curcumin-loaded collagen cryostructures have been shown to have wound-healing capabilities. Curcumin, as previously stated, has antioxidant, antibacterial, and anti-inflammatory properties. Collagen promotes cell attachment, differentiation, and migration. When curcumin is loaded directly into collagen hydrogels, it forms enormous molecular aggregates and clogs the matrix pores. Curcumin was loaded into LNPs, and these particles were embedded into collagen scaffolds using the double-encapsulation method. The resulting collagen/LNP cryostructures had an optimal fibrous structure, with an average pore size of 100 micrometers, for sustaining cell migration. The findings indicate that collagen fibers are structurally unaltered, and nanoparticles are evenly distributed. After 24 h in a saline buffer, hydrogels released 20 to 30% of their nanoparticle content, and after 25 days, they released 100%. When exposed to NIH 3T3 fibroblasts, these hydrogels provided a suitable scaffold for cell interaction as early as 4 h after seeding, with no cytotoxic side effects. Because of these advantages, collagen/lipid cryostructures are attractive materials for future wound healing applications [127]. New collagen/curcumin cryostructures were developed to retain the significant collagen matrix characteristics for wound healing, such as high microporosity, a good swelling ratio, good mechanical capabilities, and favorable cell interaction conditions. The study demonstrated that loading the drug in LNPs embedded in the cryostructures preserved all properties of the collagen scaffold, although direct curcumin loading in the collagen matrix occluded the cryostructure pores [124,127].

- Nanoparticles to Silence TNFα

The tissue factor TNFα plays a significant role in wound healing; however, in diabetic wounds, this factor can be overproduced, further damaging the wound by promoting cellular death. Small interfering RNA (siRNA) is a double-stranded non-coding RNA fragment. Since these are double-stranded, they will not code with any genomic sequence, disrupting regular sequencing and gene expression. LNPs loaded with siRNA delivered in vivo will help in silencing normal TNFα in the diabetic wound, thus helping wound healing. Animal studies were conducted using a double-wounded mouse model. One wound was treated with phosphate-buffered saline (PBS) as a negative control, while the other was treated with siRNA-loaded nanoparticles. The wound treated with these siRNA-loaded LNPs showed more rapid wound closure than that treated with phosphate buffer [39,128]. Since siRNA is large, has a negative charge, and needs a suitable vehicle, its application is challenging. So, this can be delivered with polymeric/LNPs, nanofibrous mesh, or hydrogel to the injury [39].

- Oil-Loaded LNPs

Natural products such as aloe, β-glucans, honey, and especially essential oils (EOs) have been proposed as antibacterial agents. Due to their broad spectrum of antimicrobial activity, essential oils have antimicrobial effects against numerous multidrug-resistant microorganisms. Essential oils are substances produced as secondary metabolites by plants. The plants’ nonwoody sections excrete these. Eucalyptus oil and rosemary oil are two examples. These oils are antibacterial, antifungal, and antiseptic. So, they can aid in the healing of wounds [129]. However, since they can be unstable, it is essential to prepare them in a way that enhances their biostability and antimicrobial activity. These oil-loaded NPs showed a better biocompatible pattern and proliferation properties toward fibroblast development in wound healing. Oil encapsulation inside NPs did not alter the activity while maintaining safety and efficacy [30].

- Retinoic Acid-Loaded LNPs

All-trans retinoic acid (ATRA) is described as a potential wound-healing agent but is used less frequently due to its adverse effects. Thus, specific techniques to overcome these adverse effects can be employed to use it as a better material for wound treatment. When applied to the skin surface, ATRA accelerates wound healing by boosting fibroblast proliferation in the dermis and epidermis. Nevertheless, longer contact with the skin surface can cause skin reactions. To control this, drug delivery systems can be developed to allow direct application to the wound bed. Following the inclusion of ATRA into LNPs, some polymers, such as chitosan, can be employed for regulated administration due to their low toxicity, improved biocompatibility, and biodegradable nature [130,131].

In in vivo studies, the LNP-ATRA group exhibited increased wound closure after the fifth day of wounding compared to LNP blank treatment of diabetic wounds. In addition to reducing leukocyte infiltrate in the wound bed, SLN-ATRA chitosan films increased collagen deposition and decreased scar tissue. These findings suggested that SLN-ATRA encased in chitosan films may be a good candidate for treating diabetic wounds and promoting tissue healing [131].

#### 4.6.3. Polymeric Nanoparticles

Polymeric nanoparticles typically range in size from 10 to 100 nm and have a high drug-loading capacity [132]. Discher and Eisenberg defined the configuration of polymeric nanoparticles in 2002. They claimed that it has a core-shell structure. The inner side consists of a polymeric matrix containing the hydrophobic drug, and the surface is made of a hydrophilic polymer, such as PEG or PVP, which provides steric stabilization, decreases immunogenicity, and promotes phagocytosis of nanoparticles by the reticuloendothelial system. Natural (e.g., gelatin or chitosan) or synthetic (e.g., polycaprolactone) constituent matrices and also biodegradable or nonbiodegradable polymers (for example, poly (lactic-co-glycolic acid) (PLGA) or cyanoacrylate) can be used. Biodegradable polymeric nanoparticles are favored, as they are very effective in drug delivery systems [133]. Such nanoparticles have the benefits of controlled/sustained release, subcellular scale, and biocompatibility with tissue and cells [100]. Apart from that, these nanomedicines are non-toxic, non-thrombogenic, non-immunogenic, non-inflammatory, do not stimulate neutrophils, are biodegradable, resist the reticuloendothelial cell, and can be used to deliver a wide range of molecules, including medications, proteins, peptides, and nucleic acids. Different methods for synthesizing polymeric nanoparticles can be used based on the type of drug to be incorporated into them and their conditions for a particular delivery route [37,133].

##### Chitosan Nanoparticles in Wound Healing

Necrotic tissues attract infectious bacteria, interfering with the normal wound-healing process. Autolytic debridement is a normal healing mechanism in which weak and unwanted tissues are separated from adjacent healthy tissues due to the presence of moisture. As a result, the dressing’s ability to maintain a moist atmosphere is essential for wound healing. In addition to maintaining a moist environment, the dressing should absorb excess secretions released by the wound. Infected wounds have been treated with various biopolymer-based dressings with different degrees of efficacy [49,134]. Chitosan nanoparticles were used to synthesize a biodegradable dressing. Chitosan is helpful for wound healing because of its inherent antibacterial and wound-healing effects [49]. Ionic gelation was used to prepare chitosan nanoparticles, with sodium tripolyphosphate as the crosslinker [135,136]. Although its swelling properties showed its aptitude for wound healing, the chitosan dressing demonstrated better in vitro breakdown in the presence of protease enzyme. A quantitative analysis of thrombin concentration in human blood plasma was performed using an in vitro ELISA technique dependent on the human thrombin antithrombin complex (TAT). Human dermal fibroblasts (HDFs) were used in an in vitro cell culture experiment to assess the chitosan dressing’s cell proliferation and cytotoxicity. According to the cytotoxicity assay, the synthesized dressing had no substantial cytotoxic effect against HDF cells and promoted cellular proliferation. The chitosan dressing was found to be non-toxic and to preserve the cell phenotypes of human dermal fibroblast cells using the Alamar blue biocompatibility test. The TAT assay analysis indicated that the dressing could speed up hemostasis by increasing thrombin production, making it ideal for wound dressings. These dressing characteristics can help with cellular attachment, thrombin production, increased permeability, elimination of foreign materials, and absorption of wound fluids [137,138]. Wound infections caused by multiple microbes necessitate high doses of antibiotics and fungicides, which can lead to side effects and antibiotic resistance. To address this, researchers developed chitosan bandages loaded with nanoparticles containing the antimicrobial drugs ciprofloxacin and fluconazole for treating polymicrobial wound infections. The bandages had a flexible and porous structure, allowing for the absorption of excess exudates in infectious wounds. In vitro studies showed that the bandages were not toxic to human dermal fibroblast cells and that the drugs were released over 14 days. In vitro and ex vivo, the antimicrobial bandages were effective against polymicrobial cultures of Candida albicans, *Escherichia coli*, and *Staphylococcus aureus*. In an in vivo study using a polymicrobial infected rat wound model, the antimicrobial drug-loaded chitosan bandages significantly reduced the microbial load. The findings imply that chitosan bandages with antimicrobial drug-loaded nanoparticles might be a potential therapy option for polymicrobial wound infections [139]. Another research study developed a novel wound dressing made of Spanish broom fibers impregnated with vancomycin-loaded chitosan nanoparticles. The chitosan nanoparticles were prepared using ionic gelation with varying ratios of chitosan (CH) and tripolyphosphate (TPP). The nanoparticles were evaluated for size, zeta potential, yield, encapsulation efficiency, stability, and drug release. The antibacterial activity of the nanoparticles was tested against *Staphylococcus aureus*, and the cytotoxicity to HaCaT cells was assessed. The best formulation was chosen based on encapsulation efficiency and yield. The Spanish broom fibers impregnated with nanoparticles showed increased antibacterial activity against *Staphylococcus aureus* and were not toxic to HaCaT keratinocyte cells. Overall, the Spanish broom fibers impregnated with vancomycin-loaded CH/TPP nanoparticles can be a promising wound dressing [140].

##### Gallic Acid-Loaded Chitosan Nanoparticles

Collagen has gained more consideration as a biomaterial for application because it is a key component of the extracellular matrix, is less toxic and has a biocompatible nature [141]. Collagen-based scaffolds offer a favorable moist atmosphere for tissue regeneration, preserve growth factors by reacting with them, inactivate matrix metalloproteinases (MMPs), and aid in the deposition and reorganization of de novo collagen. Collagen-based scaffolds are anti-inflammatory and analgesic, facilitate angiogenesis [142] and activate the healing cascade by changing the wounded microenvironment [141]. Collagen has several benefits, but its low mechanical strength and fast biodegradation rate hinder its use. As a result, collagen-based scaffolds with improved characteristics can be synthesized by mixing collagen with natural or synthetic polymers. In a study, ionotropic gelation was employed to synthesize a collagen-fibrin scaffold with gallic acid-loaded chitosan nanoparticles (GA-CSNPs) for wound healing. Fibrin is commonly utilized in wound and burn therapy because it helps to establish hemostasis after tissue injury. Fibrin was employed as a co-polymer with a collagen scaffold [143]. Genipin, a natural crosslinker, has been used in biological tissues and biomaterials as an efficient crosslinking agent. It is less cytotoxic and can crosslink with protein, collagen, fibrin, and other proteins [144]. Gallic acid (GA), a well-known plant polyphenolic compound, has anti-inflammatory, anti-oxidant, anti-cancer, anti-diabetic, wound healing, and other effects. GA was loaded with chitosan nanoparticles (CSNPs), which served as a safety mechanism for GA and slowed the drug’s evacuation from the wound site [145]. Drug-loaded CSNPs have a longer release time and improve wound site stabilization. The scaffold’s in vitro biocompatibility was examined using fibroblast cell lines, and an injured rat model was used to test the scaffold’s ability to promote wound healing in vivo. With an even distribution of loaded GA-CSNPs and strong swelling potential, the scaffold structure revealed its porous existence. It was also demonstrated that GA CSNPs enhanced the Col-fibrin scaffold’s capacity for radical scavenging. The GA-CSNP scaffold was tested in vitro and in vivo for biocompatibility and tissue regeneration capability. In vitro studies revealed that cells in the GA-CSNP scaffold-treated community migrated rapidly. This finding was supported by an in vivo wound healing analysis. The GA-CSNP scaffold accelerated wound contraction in laboratory rats and decreased epithelialization duration. The collagen-fibrin groups recovered more slowly, but the treated groups’ collagen and hexosamine levels improved more immediately after GA-CSNP scaffold therapy. In the GA-CSNP scaffold-treated population, histology findings revealed increased collagen deposition, angiogenesis, epithelialization, and fibroblast migration [141,143].

#### 4.6.4. Cerium Oxide Nanoparticles in Wound Healing

**Figure 4:**
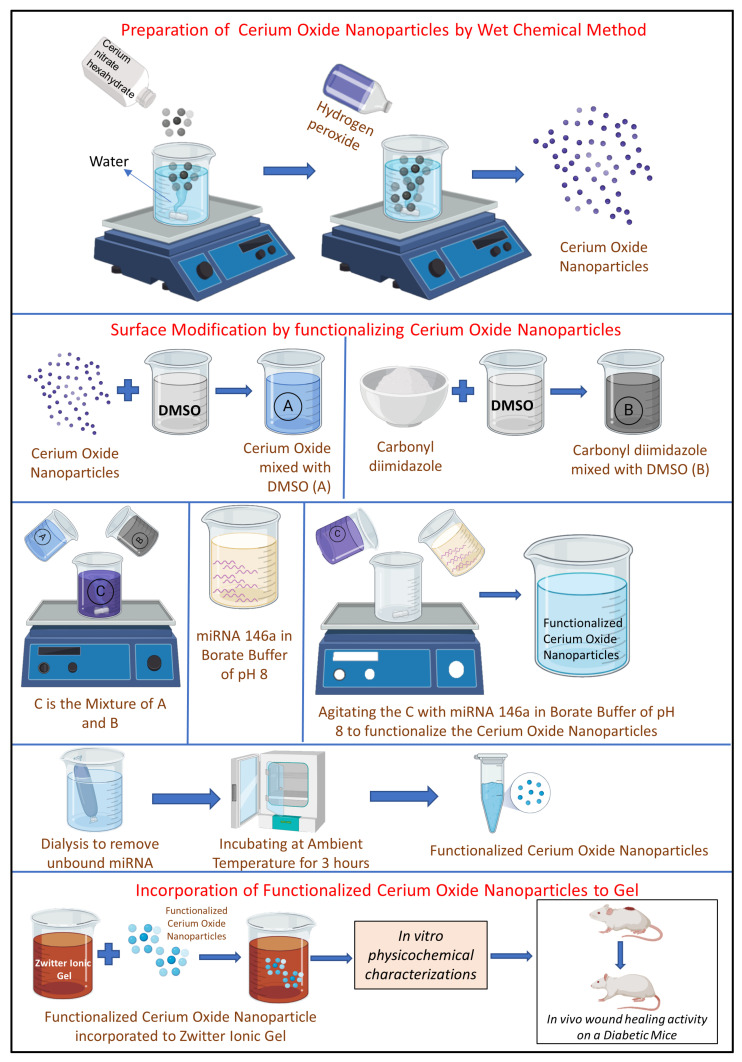
Synthesis of cerium oxide nanoparticle-impregnated gel [148]. Synthesis of cerium oxide nanoparticle using wet chemical method. Surfaces of nanoparticles were functionalized with miRNA146a. The functionalized nanoparticles were incorporated into gel, and the developed formulation was characterized.

Cerium oxide (CeO_2_) is a rare-earth metal that has recently attracted attention for biological applications due to its multi-enzyme mimetic activity and capacity to transport oxygen and scavenge free radicals through redox reactions between Ce^3+^ and Ce^4+^. Wounds typically maintain a hypoxic environment and generate free radicals, complicating healing [146,147]. This has led to an urgent need for new methods for expediting wound closure and preventing infection. Cerium oxide nanoparticles, on the other hand, can aid in overcoming these problems by scavenging free radicals, which makes them effective in lowering oxidative stress and regulating the ratio of oxidants to antioxidative enzymes in wounds. Patients with diabetes are more likely to develop chronic wounds that heal more slowly, and oxidative stress can impede healing. By utilizing zwitterionic cryogels laden with cerium oxide nanoparticles, researchers have found a way to combat this issue and improve wound healing. Additionally, as depicted in Figure 4, the nanoparticles were conjugated with microRNA-146a, which has been shown to reduce inflammation. These two components offer a potent strategy for treating diabetic patients’ chronic wounds. In testing the effectiveness of these gels, researchers found that sustained release of miRNA146a-tagged cerium oxide nanoparticles from the gels significantly reduced inflammation and improved wound healing time in a diabetic mouse model. Overall, the utilization of cerium oxide nanoparticles in this research can potentially improve wound healing outcomes for people with diabetes [148]. For treating diabetic wounds, a novel method using electrospun poly (3-hydroxybutyrate-co-3-hydroxyvalerate) (PHBV) membranes containing cerium oxide has been developed. The membrane was evaluated using in vitro cell adhesion studies, a chicken embryo angiogenesis assay, and in vivo diabetic wound healing studies. Results indicated that the CeO_2_-containing PHBV membrane promoted cell adhesion, cell proliferation, and blood vessel formation. In vivo studies confirmed the membrane’s ability to enhance the healing of diabetic lesions in rats. Therefore, the CeO_2_-incorporated PHBV membrane may serve as a promising wound dressing agent to improve the healing of diabetic wounds by promoting cell proliferation, vascularization, and wound healing [149].

Dressings made from synthetic and natural polymers provide several advantages compared to those made from a single polymer. Gelatin is a natural biopolymer derived from animal collagen through partial acid or alkaline hydrolysis. Poly (-caprolactone) (PCL) is a biodegradable and bioactive synthetic polymer extensively explored in biomedical applications; it has a wide range of applications in health sciences due to good biocompatibility and non-immunogenicity [150,151]. The use of reactive oxygen species (ROS) scavengers to promote tissue repair has been the subject of numerous experimental studies. Metal oxides, such as cerium oxide (CeO_2_), are important antioxidants that can hasten local healing by preventing the accumulation of reactive oxygen species (ROS) and reducing inflammation [152]. CeO_2_ nanoparticle-containing PCL/gelatin fibrous film was manufactured using an electrospinning technique and then tested in vivo and in vitro for its properties as a skin wound dressing medium. The characteristics of the PCL/gelatin film treated with various concentrations of CeO_2_ nanoparticles were studied on the L929 murine fibroblastic cell, and the ideal CeO_2_ dose for wound healing was identified. The results showed that the PCL/gelatin film with 1.5 percent (*w*/*v*) CeO2 nanoparticles had the most significant cell proliferation on L929 cells. The findings from in vivo studies confirmed the beneficial wound-healing effects of the CeO_2_ nanoparticle comprising film compared to sterile gauze [99,153,154]. Another research study looked into the potential of CeO_2_ nanoparticles for wound healing applications. The study involved the fabrication of a nanofiber mesh by electrospinning polycaprolactone (PCL)-gelatin blended with CeO_2_ nanoparticles and evaluated its potential for enhancing wound healing. Due to the incorporation of CeO_2_ nanoparticles_,_ the CeO_2_ nanoparticle-functionalized PCL-gelatin nanofiber mesh displayed superoxide dismutase (SOD) mimetic activity, which assisted in scavenging ROS, reducing oxidative stress, and tripling cell survival and proliferation. The mesh was also found to maintain its fibrous form for up to 14 days and to enhance the proliferation of 3T3-L1 cells by 48%. The study suggested that CeO_2_ nanoparticle-functionalized PCL-gelatin nanofibrous mesh has the potential for wound healing applications due to its ability to scavenge ROS and improve cell proliferation [155].

Cerium oxide nanoparticles were developed using an environmentally acceptable and sustainable plant-assisted synthetic method [156]. The CeO_2_ nanoparticles were synthesized in a green manner, using Zingiber officinale extract as the reducing, capping, and stabilizing agent. The synthesized nanoparticles were incorporated into a chitosan/polyvinyl alcohol hydrogel (PVA) using a simple freeze–thaw technique. The developed hydrogels of CeO_2_ nanoparticles exhibited excellent antibacterial properties against multi-drug resistant *Staphylococcus aureus* within 12 h and maintained the viability of human dermal fibroblasts for up to 5 days, with viability of more than 90%, surpassing that of the control group. The chitosan/PVA hydrogels incorporated with CeO_2_ nanoparticles have the potential to serve as an effective wound dressing agent by significantly reducing wound infections without the need for antibiotics [157].

## 5. Clinical Studies of Nanoformulations in Wound Healing

Clinical studies of nanoformulations in wound healing have shown promising results in terms of efficacy and safety. Several studies have been conducted to evaluate the use of nanomaterials in wound dressings [32]. Some of the studies have been explained in detail and tabulated in Table 1. In conclusion, clinical studies evaluating nanoformulations for wound healing have shown promising results in terms of efficacy and safety. The use of nanomaterials in wound dressings has the potential to significantly improve wound healing outcomes and reduce the risk of infection. However, further research is needed to determine these advanced wound dressings’ long-term safety and efficacy [32,158].

## 6. Future Perspectives and Scope

Nanomaterial-impregnated wound dressings have shown promising results in improving wound healing outcomes, yet their implementation in the market has been slow due to the high cost of production and the lack of regulatory approvals. Despite the increasing number of research studies, market solutions remain limited. Using nanomaterials in wound dressings increases production costs, making it difficult for manufacturers to produce them competitively, and scaling up production is also challenging. In addition, strict regulations make obtaining approval for new products lengthy and expensive, discouraging manufacturers from investing in nanomaterial-impregnated wound dressings. To address these challenges, potential solutions include improving manufacturing processes to reduce costs and working with regulatory agencies to streamline the approval process by developing specific guidelines for these wound dressings.

It is also important to consider the future perspectives and scope of improvement in this field. Novel materials hold the potential for providing real-time monitoring of wound healing and improving scalability. In addition, there is potential for these wound dressings to be applied beyond wound care, such as in regenerative medicine and tissue engineering. By collaborating with manufacturers to improve production efficiency and reduce costs, nanomaterial-impregnated wound dressings could become more widely available and affordable on the market. Furthermore, integrating these wound dressings into telemedicine platforms could provide remote monitoring of wounds and improve patient outcomes. A comprehensive awareness of the challenges in implementing nanomaterial-impregnated wound dressings in the market and exploring potential future developments and applications will broaden our understanding of the scope of this field and its potential to revolutionize wound care.

## 7. Conclusions

An injury to the tissue can be more of a challenge with a secondary microbial infection. Many internal and external factors can interrupt the physiology of the healing process, the most significant of which is microbial infection. Appropriately dressing the wound with the help of nanomaterial-impregnated wound dressings could hasten the healing of the injured tissues and prevent the wound from becoming contaminated. Nanotechnology has revolutionized the field of wound healing by providing innovative and effective ways to treat wounds. The development of nanomaterials in wound dressings has shown tremendous potential for promoting wound healing and preventing infections. Clinical studies have shown promising results, with nanoformulations demonstrating improved therapeutic effectiveness, sustained drug release, and reduced healthcare costs. It is crucial to carry out extensive research and clinical studies to prove the safety and effectiveness of nanomaterials as their application in wound care expands. Nanotechnology has enormous potential for improving wound healing and holds significant hope for the future of wound treatment. Nanoformulations have the potential to revolutionize how wounds are treated and managed, resulting in better patient outcomes, lower healthcare costs, and a higher quality of life. Overall, this review provides a comprehensive understanding of the use of nanotechnology in wound healing and underscores the importance of continued research in this area to realize the potential benefits of nanomaterials in wounds.

## Figures and Tables

**Table 1:** Clinical studies evaluating nanoformulations in wound healing.

S. No.	Title of the Study	Disease/Condition	Treatment	Detailed Description of the Study	Outcomes of the Study
1.	Evaluation of Diabetic Foot Wound Healing Using Hydrogel/Nano Silver-based Dressing vs. Traditional Dressing: A Prospective Randomized Control Study	Diabetes mellitus: diabetic foot	Hydrogel/nano silver-based dressing	The group I patients’ diabetic foot wounds were treated with a hydrogel/nano silver dressing changed every two days, while group II patients received traditional wound dressing changed once daily. After three days, the wounds were evaluated for granulation tissue, wound size, and discharge. The wound-healing process was assessed for three consecutive weeks in both groups.	The study investigated the efficacy of a hydrogel/nano silver-based dressing compared to traditional wound dressings in treating diabetic foot wounds. A total of 60 patients with type-2 diabetes and diabetic foot wounds were included in the study. The study’s results provided important insights into using hydrogel/nano silver-based dressings for diabetic foot wound management [159].
2.	Healing of burn wounds by topical treatment: A randomized controlled comparison between silver sulfadiazine and nano-crystalline silver	Burn wounds	Nano-crystalline silver	The study involved allocating patients with second-degree burn injuries to either SSD or AgNP treatment groups. Clinical assessments were carried out on the burn wounds every week until the fourth week and until the completion of treatment.	A study compared the effectiveness of silver sulfadiazine (SSD) and nano-crystalline silver (AgNP) hydrogel in managing burn wounds. AgNP has a high surface-to-volume ratio and low toxicity, making it effective at low concentrations. Results suggest that AgNP can be a superior alternative to SSD, especially for second-degree deep-dermal burns. Healing time ranges from 6 to 8 weeks, depending on body surface involvement [33].
3.	A randomized-controlled trial comparing cadexomer iodine and nanocrystalline silver on the healing of leg ulcers	Chronic leg ulcers	Nanocrystalline silver	In a randomized controlled trial, 281 community nursing clients with leg ulcers infected with bacteria were assigned randomly to receive either silver or iodine dressings for their wounds. After 12 weeks, 64% of the ulcers were observed to have healed.	Chronic leg ulcers are a debilitating and costly condition that may be aggravated by bacterial colonization, leading to infection. Although an antimicrobial dressing is clinically indicated, there is no consensus on the optimal practice for using such agents. Researchers conducted a study to compare the effectiveness of two commonly used antimicrobials, nanocrystalline silver and cadexomer iodine. The overall healing rate and the number of healed wounds were similar for both antimicrobials. However, the use of silver compounds resulted in faster healing rates during the first two weeks of treatment and in wounds that were larger, older, and had more exudate. This study provides valuable information about when one product may be preferred [160].
4.	A Healing of Chronic Wounds by Copper Oxide-Impregnated Wound Dressings—Case Series	Acute and chronic wounds	Copper oxide micro-particle-infused wound dressings	Study presented ten cases of patients with various etiologies, such as diabetes mellitus, sickle cell disease, renal failure, and necrotizing fasciitis, in which copper oxide-infused wound dressings were applied to both infected and non-infected wounds, resulting in significant improvement in wound healing. The dressings were found to clear infections, reduce fibrous and necrotic tissue, and stimulate granulation, epithelialization, and wound closure. The case reports supported the hypothesis that copper oxide-infused wound dressings not only offer protection against microbial contamination but also promote skin regeneration and wound healing.	Copper oxide micro-particle-infused wound dressings have been approved for treating acute and chronic wounds. This study aimed to provide initial evidence on the efficacy of these dressings, including their use in non-infected wounds. Patients with wounds that responded poorly or partially to traditional wound healing methods were treated with copper oxide-infused dressings. The results were compared to previous animal and in vitro studies highlighting copper’s role in skin regeneration and angiogenesis. The findings from this case series support the potential use of copper oxide-impregnated wound dressings as a valuable intervention in wound healing, particularly in cases of difficult-to-treat wounds [67].
5.	Electrospun Chitosan Nanofiber Materials as Burn Dressing	Burn	Electrospun chitosan nanofiber	Chitosan nanofiber sheets (200 μm thick) were used on 19 patients with II and IIIa burns and ten with IIIb burns pre-transplantation. Dressings were applied to donor wounds post-transplantation. The sheets maintained a moist environment for tissue regeneration, removed excess exudate, and did not cause inflammation. Thicker (400 μm) sheets were used on three patients with IIIa burns, requiring additional measures to remove excess exudate, but regeneration was similar.	A novel electrospun nanofibrous material made from biocompatible chitosan was proposed as a burn dressing. The chitosan nanofiber mats were effective in wound healing for IIIa and IIIb degree burns by absorbing exudate, preventing infection, ventilating the wound, and stimulating skin tissue regeneration. The study also explored the influence of material thickness on regenerative processes and degradation and the mechanical properties of the nanofiber mats [53].

## Data Availability

Not applicable.
